# One size does not fit all: Caste and sex differences in the response of bumblebees (*Bombus impatiens*) to chronic oral neonicotinoid exposure

**DOI:** 10.1371/journal.pone.0200041

**Published:** 2018-10-08

**Authors:** Melissa W. Mobley, Robert J. Gegear

**Affiliations:** Department of Biology and Biotechnology, Worcester Polytechnic Institute, Worcester, Massachusetts, United States of America; University of Arizona, UNITED STATES

## Abstract

Neonicotinoid insecticides have been implicated in the rapid global decline of bumblebees over recent years, particularly in agricultural and urban areas. While there is much known about neonicotinoid toxicity effects at the colony stage of the bumblebee annual cycle, far less is known about such effects at other stages critical for the maintenance of wild populations. In the present work, individual-based feeding assays were used to show that chronic consumption of the widely used neonicotinoid clothianidin at a field-realistic average rate of 3.6 and 4.0 ng/g·bee/day reduces survival of queen and male bumblebees, respectively, within a 7-day period. In contrast, worker survival was unaffected at a similar consumption rate of 3.9 ng/g·bee/day. To test the hypothesis that males have a lower tolerance for oral clothianidin exposure than workers due to their haploid genetic status, RNAseq analysis was used to compare the transcriptomic responses of workers and males to chronic intake of clothianidin at a sub-lethal dose of 0.37ng/bee/day for 5 days. Surprisingly, clothianidin consumption only altered the expression of 19 putative detoxification genes in a sex-specific manner, with 11/19 genes showing increased expression in workers. Sub-lethal clothianidin exposure also altered the expression of 40 genes associated with other major biological functions, including locomotion, reproduction, and immunity. Collectively, these results suggest that chronic oral toxicity effects of neonicotinoids are greatest during mating and nest establishment phases of the bumblebee life cycle. Chronic oral toxicity testing on males and queens is therefore required in order to fully assess the impact of neonicotinoids on wild bumblebee populations.

## Introduction

Bumblebees have rapidly declined in abundance, species richness, and geographic distribution on a global scale over recent years [1, 2]. In North America alone, nearly half of the bumblebee species have reached historically low numbers [3–5], including one species, *Bombus affinis*, which was recently listed as an endangered species by the U.S. Fish and Wildlife Service. From an ecological perspective, these declines pose a significant threat to the function and diversity of temperate ecosystems due to the critical keystone role that bumblebees play as pollinators of native flowering plants. Reports of parallel reductions in bee-pollinated plant species suggest that pollinator decline-mediated effects on wildlife diversity at higher trophic levels may already be well underway [1, 6, 7]. It is therefore imperative that all anthropogenic stressors contributing to species decline in bumblebees be identified and mitigated as soon as possible.

One stressor thought to contribute to wild bumblebee decline in urban and agricultural areas is a newly developed class of pesticides called ‘neonicotinoids’ [8]. From a pest management perspective, neonicotinoids are a highly effective form of insect control because they specifically target nicotinic acetylcholine receptors in the insect central nervous system, leading to paralysis and death [9]. However, unlike most other pesticide classes, neonicotinoids are also systemic, meaning that they are readily taken up by the roots and distributed throughout the entire plant, thereby protecting it from pest damage over the entire growing season [10].

Despite these benefits, neonicotinoids pose a significant threat to beneficial insects such as pollinators because they are translocated to floral nectar and pollen, presenting a route of oral exposure. Of particular concern to wild pollinator populations is the fact that neonicotinoids can be transported away from the area of application to adjacent natural areas [11, 12] and then contaminate wildflower resources [2, 13, 14]. For example, clothianidin, one of the newest and most potent neonicotinoid formulations, has been detected in numerous animal-pollinated wildflower species at concentrations ranging from 4 to 215 ppb [2, 14]. Compounding this issue, neonicotinoid residues can persist in the soil for years after a single application [15–17], and increase in concentration with repeated annual application [18]. Wild bumblebees and other insect pollinators therefore have the potential to be orally exposed to neonicotinoids at life stages occurring outside of the blooming period and geographic location of the targeted plant species.

**Fig 1:**
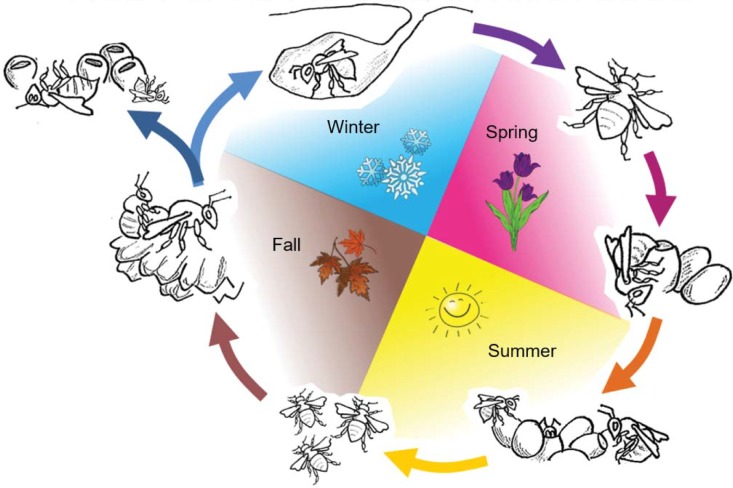
Annual cycle of social bumblebees. The cycle begins when mated queens emerge from their overwintering sites in early spring and search for suitable nest sites. Once a suitable nest site has been found, the queen collects and stores floral resources (nectar and pollen) to raise the first brood of workers, which emerges in late spring. A subset of the workers emerging become ‘foragers’ and leave the nest to collect floral resources for the queen and the rest of the workers in the colony. The number of workers (foragers and nest bees) in the colony continues to grow over the summer months. From late summer into the fall, the colony starts to produce new queens and males (reproductives) instead of workers. Reproductives leave the nest soon after emergence and search for a suitable mate until late fall. Once mated, queens search for an overwintering site and once located, reside there until the spring. Males and colonies (founding queen and workers) do not survive the winter, thus completing the cycle.

Yet, the overwhelming majority of neonicotinoid toxicity studies on bumblebees to date have focused only on the colony stage, relying on metrics such as worker survival, larval growth, and reproductive output to estimate the potential impact of exposure on wild populations [19]. While such colony-focused risk assessment protocols may be adequate in the context of crop pollination, determining the impact of widespread neonicotinoid use on threatened bumblebee species in an ecological context requires more comprehensive assessment protocols that consider potential impacts on queens and males, whose survival during mating, overwintering, and nest establishment stages of the life cycle has a direct effect on population stability (Fig 1).

Several morphological and physiological characteristics of queen, worker, and male bumblebees suggest that individuals from each group may vary in their capacity to cope with chronic oral neonicotinoid exposure. For example, males and workers have a smaller body size than queens and therefore may experience mortality effects at lower neonicotinoid concentrations, as has been shown at the species level [20]. Males are also haploid whereas queens and workers are diploid, which may limit gene products available for males to detoxify neonicotinoids. Such ‘haploid susceptibility’ is known to occur in the context of immunocompetence [21], but has yet to be studied in the context of metabolic resistance to pesticides. Alternatively, males and queens have greater energetic demands due to their reproductive status [22, 23] and therefore may have fewer resources available for detoxification processes than sterile workers.

To explore these possibilities, the present study uses a novel individual-based feeding assay to directly compare the mortality response of queen, worker, and male *Bombus impatiens* to chronic consumption of the widely used neonicotinoid clothianidin at field-realistic concentrations of 5-10ppb. Assaying bumblebee test populations at the individual level provides a much more robust estimate of chronic lethality level than more traditional assays on small groups (e.g. microcolonies) because drug intake rates can be directly monitored in all test individuals and then easily standardized by adjusting for individual variation in body size and daily intake [24].

The results of our initial feeding experiments revealed that chronic lethality effects of clothianidin are much greater in males than workers even when controlling for differences in body size. To test the potential role of male haploidy in driving this sex-specific response to clothianidin exposure, we then used RNAseq analysis to compare the transcriptomic responses of workers and males to consumption of clothianidin at a sub-lethal daily dose over a 5-day period. Following previous work on bumblebees by [25], putative genes related to clothianidin detoxification were identified and classified based on the two well-characterized xenobiotic detoxification phases in insects, with Phase 1 processes metabolizing the xenobiotic to less toxic compounds through oxidation, hydrolysis, and reduction reactions, and Phase 2 processes breaking down the metabolites produced from Phase 1 processes through conjugation reactions. Based on the findings of [25] that another bumblebee species (*B*. *huntii*) has fewer constitutively expressed detoxification genes than workers, it was predicted that male *Bombus impatiens* would have fewer and/or lower expression of inducible detoxification genes compared to workers.

## Materials and methods

### Experimental design

#### Bumble bees

Virgin queens, workers, and males were obtained from commercial *Bombus impatiens* colonies (Biobest Biological Systems, Leamington, Canada). Colonies initially contained approximately 100 workers and were subsequently supplied with 30% (stock) sugar solution (Grade A pure honey mixed with distilled water to the desired concentration, measured with a Bellingham & Stanley hand-held refractometer (Suwanee, USA)) and wildflower pollen *ad libitum* to facilitate continued production of workers and later production of queens and males. Multiple colonies were used across experimental trials to control for potential inter-colony differences in clothianidin sensitivity.

**Fig 2:**
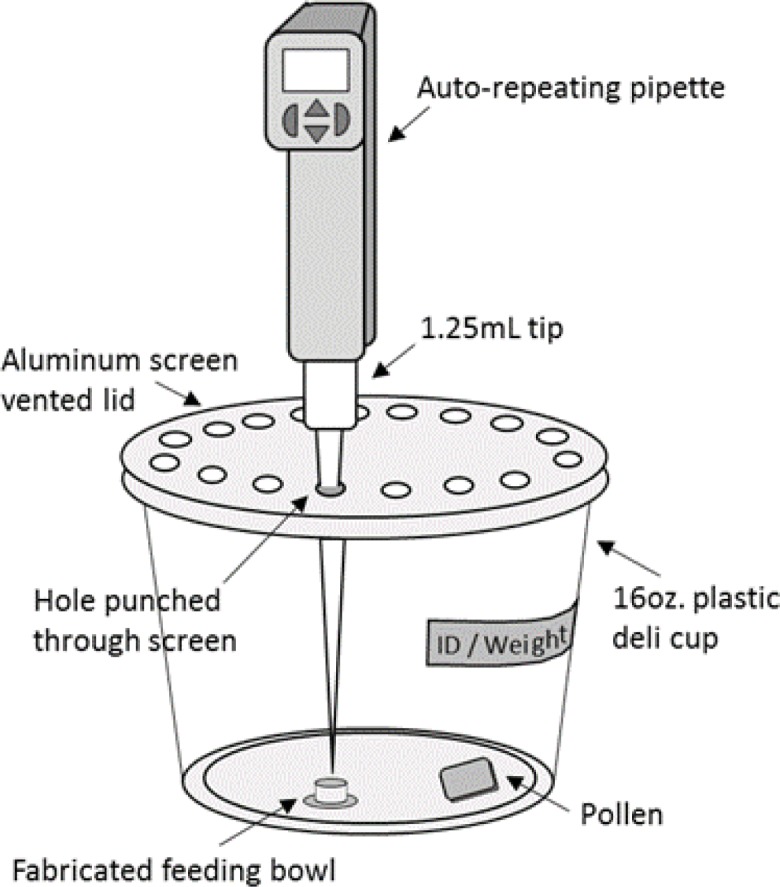
Schematic of setup for individual feeding assay. Bees were isolated from the colony and housed in a 16oz plastic container with a feeding cup positioned directly under an open vent in the screen lid. Test sugar solutions were delivered through a hole in the lid with a micropipette. Individuals were supplied with pollen *ad libitum*.

For chronic oral toxicity tests, queen, worker and male bees were collected and placed in a 4˚C refrigerator. Once immobile, individuals were weighed to the nearest milligram, marked with acrylic paint for identification, and then placed in a 16oz plastic housing container with a screen lid for ventilation (Fig 2).

Each lid had a circular opening with a removable plastic plug that was positioned directly above a plastic ‘feeding bowl’ fastened to the bottom of the container. In this way, test solutions could be dispensed into the bowls with minimal disturbance to the bee. Each container was also supplied with 1–1.5 grams of a pollen paste (a dough-like mixture of and stock sugar solution), which was replaced as necessary.

All housing containers were kept in Percival Scientific environmental chambers (Perry, USA) set to 22–25˚C, 50% humidity, and a 12 hour light/dark cycle. Workers and males were fed 75 μL and queens were fed 200 μL of untreated stock sugar solution once daily within 3 hours of the start of the light cycle. Preliminary experiments with stock sugar solution determined that these volumes are completely consumed by individuals over a 24-hour period while having no effect on survival or activity level. All handling of housing containers was conducted under red light in order to minimize additional stress on test bees.

#### Clothianidin solutions

Analytical-grade clothianidin (Sigma-Aldrich, USA) was dissolved in dimethyl sulfoxide (DMSO), as has been done in other neonicotinoid toxicity studies [26], to a concentration of 5 x 10^6^ ng/mL or ppb (parts per billion) and serially diluted down to 5 x 10^2^ or 5 x 10^1^ ppb. Diluted clothianidin solution was added to stock sugar solution to concentrations of 10, 7, and 5 ppb (ng/mL). These concentrations of clothianidin were selected because they are well within the range found under field conditions [27]. Drug mixtures were prepared within 2 days of the start of a trial, stored in conical tubes in the dark at 4˚C and only taken out for immediate use.

To confirm that test solutions contained the correct amount of clothianidin, fresh samples of 0 (stock sugar solution alone), 5, 7, and 10 ppb test solutions were sent to the U.S.D.A. Agricultural Marketing Service’s National Science Laboratories Testing Division (Gastonia, USA) for analysis. Clothianidin residues were measured using Mass Spectrometry with a LOQ of 5 +/- 1 ppb. Although the 5ppb test solution was below the minimal level of detection by the U.S.D.A. equipment, 7 and 10 ppb test solutions (3 technical replicates each) yielded mean values (+/-SE) of 4.1 +/- 3.5 and 7.3 +/-1.4 ppb, respectively.

### Test procedure for feeding experiments

Prior to each trial, all bees were fed stock sugar solution at the previously described volumes daily until there were no observed deaths over a 48-hour period. Individuals not consuming all of the sugar solution within a 24 hour period were removed.

After the 48-hour acclimation period, individuals were assigned to either the 0 ppb (control), 5 ppb, 7ppb, or 10ppb treatment group, with colony origin and bee size balanced among groups. Location of housing containers within and between shelves inside the environmental chamber was randomized in order to control for potential positional effects on survival. Total number of bees tested at each clothianidin concentration was as follows: 208 at 0ppb (88 workers, 58 queens, 62 males), 178 at 5ppb (70 workers, 58 queens, 50 males), 178 at 7 ppb (70 workers, 58 queens, 50 males), and 264 at 10 ppb (108 workers, 84 queens, 72 males). These numbers were obtained through 11 independent worker, 8 queen, and 12 male trials, with bees in each trial obtained from at least two colonies. Given queens required a greater volume of test solution than workers and males (200 μL vs 75 μL, respectively) to remain active than workers and males due to their larger body size, we standardized exposure level for each group by expressing it as ng clothianidin consumed per gram of bee per day (ng/g·bee/day). Daily intake for queens = 2 ng/bee (10ppb), 1.4 ng/bee (7ppb), 1 ng/bee (5ppb); workers and males = 0.75 ng/bee (10ppb), 0.525 ng/bee (7ppb), and 0.375 ng/bee (5ppb). Mean (±SD) mass in grams of queens = 0.58±0.12 g; workers = 0.15±0.05 g; males = 0.14±0.04 g. In this way, we could directly test for sex and caste differences in clothianidin sensitivity.

Each day over a 7-day testing period, individual housing containers were removed from the environmental chamber within three hours after to the beginning of the light cycle, and the number of dead bees was recorded. In the rare case that a test individual was not dead but showed inverted orientation, extremity spasms, and unresponsiveness, it was recorded as dead and euthanized. Presence/absence of test solutions was also checked daily and feeding bowls were replenished when necessary. Immediately following data collection, bees were returned to the environmental chamber.

To confirm that consumption of dimethyl sulfoxide (DMSO) at the concentration used in experiments had no effect on survival of queens, workers and males, additional feeding trials were conducted with stock sugar solution containing 1.4% DMSO. As in the clothianidin test trials, workers and males were fed 75 μL of solution per day and queens were fed 200 μL/day over a 7-day period. Survival in the control (0ppb) group (see above for numbers in each group) was then compared to survival in the 1.4% DMSO group for queens (24 treated individuals), workers (55 treated individuals), and males (87 treated individuals). Results of Mantel-Cox Log-rank analyses revealed that consuming 1.4% DMSO for seven days had no effect on survival of queens (X^2^ = 0.61, df = 1, P = 0.43), workers (X^2^ = 0.26, df = 1, P = 0.60) and males (X^2^ = 0.08, df = 1, P = 0.77).

#### Statistical analysis

Survival data from queens, workers, and males were compiled for the full 7-day trial period. All remaining survivors were censored. For each clothianidin concentration, Kaplan-Meier survival curves [28] were generated for queens, workers, and males in GraphPad Prism 6 (La Jolla, USA) and a Mantel-Cox Log-rank analysis was used to test for differences in survival between treatment groups and when significant, survival between a single clothianidin concentration and the 0ppb control.

### Test procedure for RNAseq analysis

To test the hypothesis that males in our feeding experiments showed greater mortality rates than workers because they have fewer inducible gene products available to detoxify clothianidin than workers (haploid susceptibility), RNAseq analysis was used to conduct genome-wide transcriptome analysis of workers and males after consumption of 75uL of either the 5ppb test solution (0.374ng/bee; 3 workers and 3 males; Test group) or 1.4% DMSO in stock sugar solution (3 workers and 3 males; Vehicle Control group) once a day for 5 consecutive days. This clothianidin dose was selected because it did not reduce worker and male survival in our feeding experiments. This approach allowed us to isolate changes in gene expression induced by clothianidin consumption from changes induced by the consumption of DMSO, which is a xenobiotic and therefore has the potential to induce expression of genes involved in detoxification processes.

#### Treatment and sample preparation

Individuals were euthanized at -20˚C 24 hours after the last dose (day 6), immediately dissected on ice by removing legs, wings, and proboscis, and transferred to -80˚C until ready for extraction. (This entire process was performed as quickly as possible to minimize RNA degradation, usually completed within 5 minutes.) Individual sample extractions were completed in an RNA-specified biosafety cabinet by crushing the bee body through pestle homogenization in Trizol *(*ThermoFischer Scientific, USA) following the suppliers recommendations for RNA isolation followed by chloroform phase separation. The aqueous phase containing RNA was collected, then purified using a PureLink RNA Mini Kit *(*ThermoFischer Scientific, USA*)*, ending with a 30 uL elution step. Concentration and purity (A260/280 and A260/230) were measured using Nanodrop. Purified RNA was stored at -80˚C until use.

#### RNA sequencing

Purified RNA samples were diluted to 100ng/uL in sterile, DNase-free, RNase-free water and then shipped to Quick Biology (Pasadena, USA) for processing. Libraries for RNA-Seq were prepared with a KAPA Stranded RNA-Seq Kit. The workflow consisted of mRNA enrichment, cDNA generation, and end repair to generate blunt ends, A-tailing, adaptor ligation and PCR amplification. Different adaptors were used for multiplexing samples in one lane. Sequencing was performed on Illumina Hiseq3000/4000 for a pair end 150 run. Data quality check was done on an Illumina SAV (San Diego, USA). De-multiplexing was performed with Illumina Bcl2fastq2 v 2.17 program. Reads were first mapped to the latest UCSC transcript set using STAR version 2.4.1d and the gene expression level was quantified using an E/M algorithm in Partek Genomics Suite (Partek, Inc. USA). Gene expression levels were normalized to total counts.

All four experimental groups produced a similar number of mean total reads: worker controls 4.951*10^6^ ± 1.845*10^5^, worker clothianidin 5.147*10^6^ ± 4.004*10^5^, male controls 5.520*10^6^ ± 8.654*10^4^, and male clothianidin 5.889*10^6^ ± 1.168*10^5^. Reads were aligned to the BIMP_2.0 genome [29], resulting a high mean percentage of alignment in all four groups: worker control 73.20% ± 3.35, worker clothianidin 80.41% ± 1.573, male control 79.15% ± 1.511, male clothianidin 77.58% ± 1.938, corresponding with 15,896 identified genes. RNA sequencing data quality and alignment for sex and treatment are shown in S1 Table.

#### Statistical analysis

Normalized gene expression levels were compared between groups using the differential gene expression algorithm (GSA) in Partek Flow (Partek, Inc., USA). GSA identifies the best statistical model among many that is the best for a specific gene, and then uses it to calculate p-value and fold change. All genes induced by clothianidin consumption were first isolated separately for each sex by comparing gene expression levels Test and Vehicle Control groups separately. In this initial screening process, a gene was considered to be differentially expressed if it had both a p-value (uncorrected) less than 0.05 and a fold change greater than 1.5. All differentially expressed genes in workers and males were then pooled to create a list of clothianidin-sensitive candidate genes.

To test for sex differences in the number and expression of detoxification genes induced by oral exposure to clothianidin, GSA analysis in Partek Flow was then used to compare normalized read counts for each clothianidin-sensitive candidate gene between workers and males in the Test group. A similar comparison of workers and males from the Vehicle Control group was not performed because there was no oral exposure to clothianidin. For this comparison, a false-discovery rate (FDR) approach was used to correct for multiple testing [30]. Genes with FDR step up values (corrected p-values) less than 0.1 were deemed to be significantly different. Each differentially expressed gene was then categorized as being involved in either Phase I (oxidation, hydrolysis and reduction) or Phase II (conjugation with sugars, glutathione, amino acids, etc.) detoxification or assigned to some other biological process based on information from the UniProt online gene database (http://www.uniprot.org/). This method of identifying and categorizing putative detoxification genes has been used previously in bumblebees and other insects [25] and [31]. A heat map of all differentially expressed detoxification genes was generated using the online web tool ClustVis (http://biit.cs.ut.ee/clustvis/). In ClustVis, the Eucledian method was applied for clustering on gene and sample sets.

## Results

### Chronic oral toxicity effects of clothianidin differ among queen, worker, and male bumblebees

**Fig 3:**
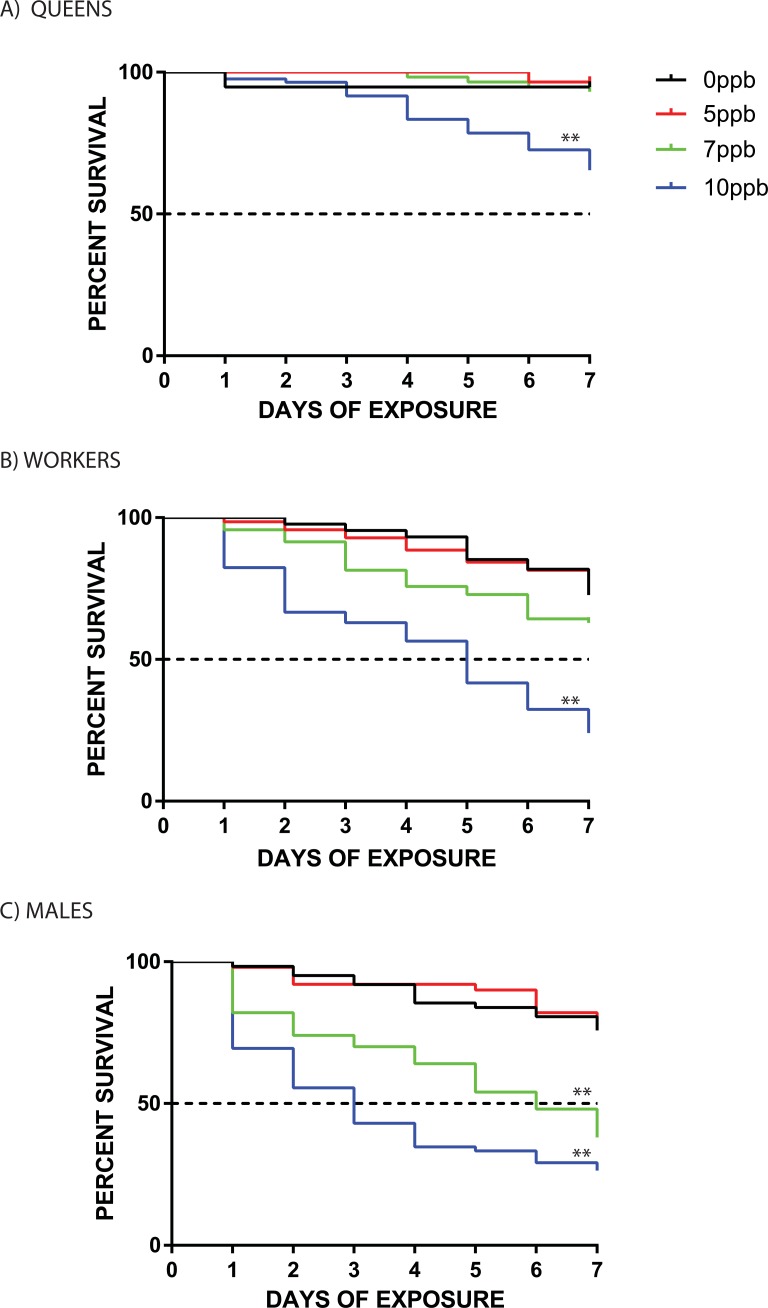
**Kaplan-Meier survival plots for *Bombus impatiens* (A) queens, (B) workers, and (C) males, orally exposed to test solutions containing different concentrations of clothianidin daily for 7 days.** Black = 0ppb; red = 5 ppb; green = 7 ppb; blue = 10ppb. A Mantel-Cox Log-rank analysis was used to test for differences in survival between each treatment group relative to the 0ppb control group over the 7-day period. **, p<0.0001.

Chronic consumption of clothianidin at field-realistic concentrations increased mortality rates in all test bumblebee populations (Fig 3); however, the chronic exposure level needed to produce such mortality effects varied in a sex and caste dependent manner. Table 1 shows the responses of queen, worker, and male test populations to daily consumption of clothianidin controlling for differences in body size and daily volume of test solution consumed. Compared to untreated controls, queens consuming clothianidin at mean intake rate ± SD = 3.61±0.71 ng/g·bee/day had reduced survival over the 7-day test period (Fig 3; X^2^ = 15.6, df = 1, P<0.0001). In contrast, workers showed reduced survival at mean intake rate ± SD = 5.48±1.95 ng/g·bee/day (Fig 3; X^2^ = 54.7, df = 1, P<0.0001), with the test population reaching 50% mortality at day 5. Interestingly, although an intake rate of 3.61 ng/g·bee/day was sufficient to reduce queen survival, a similar intake rate of of 3.85 (±1.6) ng/g·bee/day (Table 1) had no effect of worker survival, indicating that workers are better able to cope with chronic oral clothianidin exposure than queens.

**Table 1:** Response of queen, worker, and male bumblebees (*Bombus impatiens*) to chronic clothianidin exposure controlling for differences in body size and daily dose. Workers and males consumed 75 μL of solution per day and due to their larger size, queens consumed 200 μL of each solution per day. Yellow shading = no effect on survival; blue shading = reduced survival. All values are shown as mean ± SD.

	Concentration of clothianidin solution					
	5ppb(ng/mL)		7ppb(ng/mL)		10ppb(ng/mL)	
	Body mass (g)	Intake rate (ng/g.bee/day)	Body mass(g)	Intake rate (ng/g.bee/day)	Body mass (g)	Intake rate (ng/g.bee/day)
**Queens**	0.58±0.13	1.79±0.39	0.59±0.11	2.45±0.48	0.57±0.11	3.61±0.71
**Workers**	0.16±0.05	2.60±0.83	0.15±0.04	3.85±1.6	0.15±0.04	5.48±1.95
**Males**	0.14±0.04	2.94±0.86	0.14±0.04	4.01±1.08	0.13±0.03	6.05±1.35

Similar to queens a mean intake rate ± SD = 4.01 ±1.08 ng/g·bee/day was sufficient to reduce survival in males (Fig 3; X^2^ = 38.8, df = 1, P<0.0001), with test populations reaching 50% mortality after 6 days. Virtually all surviving queens, workers, and males in test groups fully consumed the clothianidin solutions over the 7-day period (S2 Table), indicating that the observed caste and sex differences in clothianidin-induced mortality level were caused by drug toxicity rather than starvation (i.e., bees refusing to consume sugar solutions because they were distasteful).

### Sex differences in the transcriptomic response of bumblebees to sub-lethal doses of clothianidin

**Fig 4:**
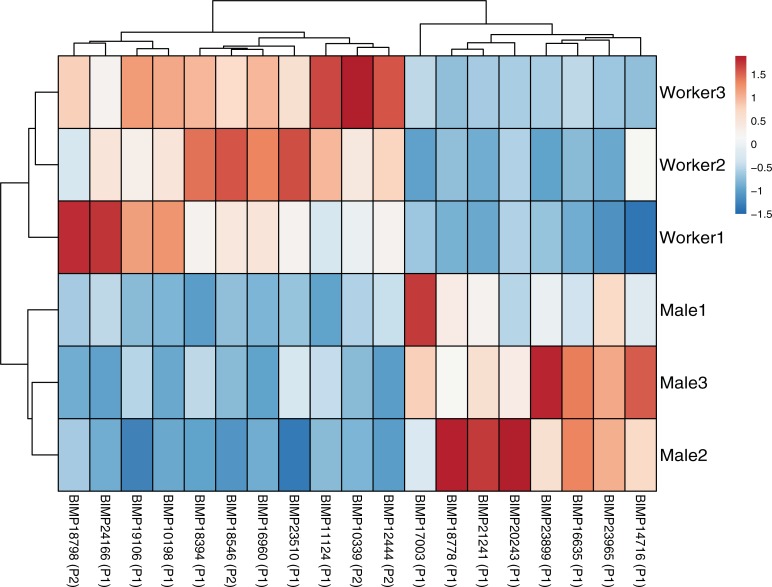
Heatmap showing the 25 putative detoxification genes that were differentially expressed between workers and males after 5 consecutive days of oral exposure to clothianidin. Phase 1 (P1) detoxification processes include oxidation, hydrolysis, and reduction reactions while Phase 2 (P2) processes include conjugation reactions. Legend on the right shows relative fold change corresponding to each color.

Initial comparisons of gene expression levels between Test and Vehicle Control groups within each sex yielded a total of 147 genes in workers and 200 genes in males that met our differential expression criterion of p <0.05 and a 1.5 or greater fold change. Of these 347 genes 15 were present in both sexes, yielding a total of 332 unique clothianidin-inducible genes. Only 59 of the 332 clothianidin-inducible genes were differentially expressed between workers and males in the Test group based on an FDR cut-off of 0.1: 34 genes had higher expression in exposed workers and 25 genes had higher expression in exposed males. Of these 59 genes, 19 were associated with detoxification processes: 11 had higher expression in exposed workers (7 Phase 1 and 4 Phase 2) and 8 had higher expression in exposed males (8 Phase 1 and 0 Phase 2; Table 2; Fig 4).

**Table 2:** Sex-specific expression of inducible detoxification genes in bumblebee workers and males orally exposed to clothianidin at a sub-lethal daily dose over 5 consecutive days. Phase 1 detoxification processes include oxidation, hydrolysis, and reduction reactions while Phase 2 processes include conjugation reactions. Expression levels were determined through RNAseq analysis. Upper portion = genes with increased expression in workers; lower portion = genes with increased expression in males. Clothianidin-inducible genes were initially identified by comparing transcriptomic expression patterns between individuals fed either 5ppb clothianidin solution or the same volume of solution containing 1.4% DMSO (vehicle control) solution separately for each sex and then pooling genes with significantly altered expression together.

**DETOXIFICATION GENES: INCREASED EXPRESSION IN WORKERS VERSUS MALES**				
**Gene ID**	**P**^a^	**Fold increase**	**Annotation**	**Phase**
BIMP10198	0.000006	6.07	cytochrome P450 6k1-like	1
BIMP19106	0.03	1.84	fatty-acid amide hydrolase 2-like	1
BIMP23510	0.04	2.45	cytochrome P450 306a1	1
BIMP18394	0.06	1.69	N-sulphoglucosamine sulphohydrolase	1
BIMP24166	0.07	1.71	U6 snRNA phosphodiesterase	1
BIMP11124	0.09	1.63	probable cation-transporting ATPase 13A3	1
BIMP16960	0.09	1.49	retinol dehydrogenase 12-like	1
BIMP12444	0.005	3.12	hydroxymethylglutaryl-CoA synthase 1	2
BIMP10339	0.02	3.25	probable serine/threonine-protein kinase yakA	2
BIMP18546	0.02	2.12	putative glycerol kinase 5	2
BIMP18798	0.09	1.67	probable deoxyhypusine synthase	2
**DETOXIFICATION GENES: INCREASED EXPRESSION IN MALES VERSUS WORKERS**				
**Gene ID**	**P**^a^	**Fold increase**	**Annotation**	**Phase**
BIMP23965	0.005	5.65	lysozyme 2-like	1
BIMP21241	0.006	4.86	phospholipase A2-like	1
BIMP18778	0.04	1.86	endoglucanase 19-like	1
BIMP17003	0.04	3.47	cytochrome P450 6k1-like	1
BIMP20243	0.049	5.60	alkaline phosphatase 4-like	1
BIMP16635	0.06	3.97	probable cytochrome P450 304a1	1
BIMP14716	0.08	1.80	venom acid phosphatase Acph-1-like	1
BIMP23899	0.09	1.62	sarcosine dehydrogenase mitochondrial	1

^a^FDR adjusted p-value

Of the remaining 40 genes associated with biological functions other than detoxification, 23 had higher expression in workers and 17 had higher expression in males (S3 Table). Biological functions, fold changes, and accession numbers for these 40 genes are shown in S3 Table. Although many of these genes may play some as yet unknown functional role in detoxification, currently known biological functions include immunity, learning and memory, reproduction, and signal transduction. We also found 12 genes whose function we could not identify based on current databases. Information for the 273/332 clothianidin-inducible genes with conserved expression between workers and males is provided in S4 Table.

## Discussion

The present study shows that chronic consumption of field-realistic doses of the widely used neonicotinoid pesticide clothianidin has differential effects on the survival of queen, worker, and male bumblebees. Controlling for body size, queens and males consuming clothianidin at an average intake rate of 3.6 and 4.0 ng clothianidin/g·bee/day, respectively, had reduced survival over a 7-day period whereas a similar average consumption rate in workers (3.9 ng/g·bee/day) had no mortality effect. However, only male test populations reached 50% mortality at this clothianidin intake rate (Fig 2) suggesting that they are slightly more sensitive to clothianidin toxicity effects than queens. The observed sex and caste differences in chronic oral toxicity of clothianidin cannot be attributed to a starvation effect as food avoidance and vomiting behaviors were not observed during the testing period. Collectively, these findings demonstrate that bumblebee reproductives (queens and males) are much more vulnerable to clothianidin toxicity effects than workers, highlighting the importance of incorporating queen and male survival assays into current colony-focused ‘higher tier’ protocols for assessing the potential risk of various neonicotinoid formulations to wild bumblebee populations [19]. In addition, the observed reductions in survival in *B*. *impatiens* workers chronically consuming clothianidin at a concentration of 10ppb is notably less than the 20ppb chronic lethality threshold reported for worker honeybees [32] as well as the 25ppb threshold reported for workers of the European bumblebee species *B*. *terrestris* [33], indicating that chronic oral lethality effects of clothianidin are relatively high in *B*. *impatiens*. Our findings thus add to the rapidly growing body of evidence that neonicotinoid oral toxicity effects can vary considerably among insect pollinator species [34–38], and further show that toxicity effects can vary considerably within species as well.

In addition to increasing mortality, chronic oral exposure to clothianidin over a short time period (5 days) was found to have profound sub-lethal effects on workers and males at the genomic level, altering expression of a total of 332 genes associated with a wide variety of biological functions, including immunity, neuronal signal transduction, locomotion, reproduction, and several fundamental cellular processes (Table 2, S3 and S4 Tables). These results are consistent with several studies at the organismal level showing that neonicotinoids can have substantial sub-lethal effects on bees [39–43]. For example, we found that neonicotinoid consumption induced changes to several genes associated with reproduction such as outer dense fiber protein 3-like and lutropin-choriogonadotropic hormone receptor-like (S3 Table), which is consistent with previous work showing that oral exposure to neonicotinoids reduces sperm viability in males honeybees by 50% [44]. Under natural conditions, chronic exposure to low doses of clothianidin in wildflower nectar thus has the potential to negatively impact the dynamics of wild bumblebee populations by reducing male and queen reproductive capacity and at slightly higher doses, reducing the number of mating individuals in the fall and the number of queens establishing nests in the spring.

Somewhat surprisingly, the genomic response of workers and males to sub-lethal clothianidin doses did not provide unequivocal evidence that the haploid genetic status of males renders them less able to cope with oral clothianidin exposure. Indeed, males and queens, which are diploid, responded similarly to clothianidin consumption in our feeding experiments (Table 1). Of the 332 clothianidin-sensitive genes that were identified, 273 had a similar expression level in workers and males. These similarly expressed genes included many ‘classic’ detoxification genes such as cytochrome P450s, glutathione S-transferases, and carboxylesterases [45]. There was, however, some support for the haploid susceptibility hypothesis for the 19 clothianidin-induced detoxification genes found to differ between workers and males. Compared to males, workers had higher expression of all differentially expressed genes associated with Phase 2 conjugation reactions. This finding, combined with the finding that workers and males had a similar number of highly expressed Phase 1 genes related to oxidation-reduction reactions, suggests that are were equally capable of initially breaking down clothianidin into intermediate metabolites, but may differ in the capacity to subsequently transform such metabolites into less toxic compounds (Phase 2 reactions). This interpretation is consistent with reports that workers have more than twice as many constitutively expressed conjugation genes than males in a related bumblebee species, *B*. *huntii* [25]. However, it must be noted that *B*. *huntii* workers had more than twice as many constitutively expressed Phase 1 genes than males as well. Thus, despite the fact that there was a substantial difference in the mortality response of workers and males to oral clothianidin exposure, it was not strongly correlated with the number and expression of inducible detoxification genes. Of course it is possible that haploidy in males results in the reduced expression of few key genes in the clothianidin detoxification pathway. For example, clothianidin-exposed workers showed a more than 6-fold increase in the expression of a cytochrome P450 6k1-like gene (Table 1), which may play an integral role in neonicotinoid detoxification. We plan to explore this possibility in future work.

The present study demonstrates the potential conservation benefits of using a genomic approach to investigate the effects of extremely low doses of neonicotinoids on pollinator health, an idea that has been recently advocated by others [46]. Currently, one of the major problems with studying neonicotinoid effects on wild bee populations is that individual tissue samples have concentrations that are too low to be quantified with a high level of accuracy and reliability using conventional methodology and instrumentation. Indeed, the main laboratory at the United States Department of Agriculture responsible for measuring neonicotinoid levels in tissue samples was unable to detect clothianidin in our 5ppb in test solution, yet it produced a robust genetic response in both males and workers. To circumvent this problem, we have identified a set of clothianidin-induced genes in bumblebees that could potentially be used to develop highly sensitive ‘eco-tools’ for assessing neonicotinoid exposure levels under field conditions. Such tools could also be used to test for sub-lethal effects on behavior (e.g. reduced cognitive function) and physiology (e.g. reduced sperm production), which are often difficult to assess in the field at the organismal level. Comparative genomic approaches would also provide unique insight into why chronic oral toxicity levels for neonicotinoids vary considerably among (and within) wild bees species [37], greatly accelerating efforts to determine the impact of neonicotinoid use on wild pollinators and the global biodiversity that they ultimately support.

## Supporting information

S1 Table RNA sequencing results and quality control. (DOCX)

S2 Table Percent of alive queens, workers and males consuming test solutions (clothianidin concentration given in ppb) over the 7-day testing period. (DOCX)

S3 Table List of clothianidin-induced non-detoxification genes with increased expression in worker (W) and male (M) *Bombus impatiens*. (DOCX)

S4 Table List of clothianidin-induced genes showing a similar expression level in worker (W) and male (M) *Bombus impatiens*. (DOCX)
