# Supplementary material for: One size does not fit all: Caste and sex differences in the response of bumblebees (Bombus impatiens) to chronic oral neonicotinoid exposure
Source: PLoS One. 2018 Oct 8;13(10):e0200041. doi: 10.1371/journal.pone.0200041 (PMC6175506; doi:10.1371/journal.pone.0200041)
Supplement: S1 Table — (DOCX) [file pone.0200041.s001.docx]

**S1 Table. RNA sequencing results and quality control.** CLO = individuals consuming 75uL of sugar solution containing clothianidin at a concentration of 5ppb daily for 5 consecutive days; Control = individuals consuming the same volume of vehicle control solution over the same time period.

| **Sample ID** | **Total reads** | **Total alignments** | **Aligned** | **Unique singleton** | **Unique paired** | **Non-unique paired** | **Non-unique singleton** | **Coverage** | **Avg. coverage depth** | **Avg. length** | **Avg. quality** | **%GC** |
| --- | --- | --- | --- | --- | --- | --- | --- | --- | --- | --- | --- | --- |
| Female Control 1 | 4,975,390 | 8,046,116 | 79.09% | 0% | 78.01% | 1.09% | 0% | 19.24% | 24.45 | 150.43 | 38.02 | 40.62% |
| Female Control 2 | 4,619,813 | 6,423,429 | 67.48% | 0% | 66.19% | 1.29% | 0% | 18.97% | 19.72 | 150.43 | 38.18 | 41.22% |
| Female Control 3 | 5,257,591 | 7,948,230 | 73.03% | 0% | 71.62% | 1.40% | 0% | 19.49% | 23.78 | 150.43 | 38.23 | 42.17% |
| Female CLO 1 | 4,361,450 | 7,004,718 | 77.32% | 0% | 76.00% | 1.32% | 0% | 18.78% | 21.83 | 150.43 | 38.22 | 41.52% |
| Female CLO 2 | 5,403,722 | 9,108,226 | 82.47% | 0% | 81.40% | 1.07% | 0% | 20.47% | 26.13 | 150.44 | 38.28 | 40.66% |
| Female CLO 3 | 5,675,210 | 9,466,214 | 81.44% | 0% | 80.32% | 1.12% | 0% | 20.15% | 27.57 | 150.42 | 38.03 | 41.70% |
| Male Control 1 | 5,650,562 | 8,920,097 | 76.66% | 0% | 75.43% | 1.24% | 0% | 20.85% | 25.01 | 150.44 | 38.25 | 40.34% |
| Male Control 2 | 5,553,565 | 9,262,420 | 78.92% | 0% | 77.31% | 1.61% | 0% | 20.75% | 26.18 | 150.43 | 38.24 | 40.88% |
| Male Control 3 | 5,356,400 | 8,966,711 | 81.88% | 0% | 80.76% | 1.12% | 0% | 20.38% | 25.76 | 150.44 | 38.24 | 40.78% |
| Male CLO 1 | 6,093,224 | 9,565,884 | 76.46% | 0% | 75.28% | 1.18% | 0% | 20.79% | 26.94 | 150.44 | 38.22 | 40.94% |
| Male CLO 2 | 5,688,606 | 8,999,731 | 74.92% | 0% | 73.35% | 1.57% | 0% | 20.43% | 25.75 | 150.44 | 38.25 | 41.11% |
| Male CLO 3 | 5,886,178 | 9,885,521 | 81.35% | 0% | 80.05% | 1.30% | 0% | 19.14% | 30.29 | 150.43 | 38.27 | 42.31% |
