# Supplementary material for: One size does not fit all: Caste and sex differences in the response of bumblebees (Bombus impatiens) to chronic oral neonicotinoid exposure
Source: PLoS One. 2018 Oct 8;13(10):e0200041. doi: 10.1371/journal.pone.0200041 (PMC6175506; doi:10.1371/journal.pone.0200041)
Supplement: S2 Table — (DOCX) [file pone.0200041.s002.docx]

**S2 Table. Percent of surviving queens, workers and males consuming full amounts of test solutions (clothianidin concentration given in ppb) over the 7-day testing period.**

| **Treatment** | **Queens** | | **Workers** | | **Males** | |
| --- | --- | --- | --- | --- | --- | --- |
|  | **Day** | **Percent consuming** | **Day** | **Percent consuming** | **Day** | **Percent consuming** |
| **0ppb** | **0** | 100.00 | **0** | 100.00 | **0** | 100.00 |
| **5 ppb** |  | 100.00 |  | 100.00 |  | 100.00 |
| **7 ppb** |  | 100.00 |  | 100.00 |  | 100.00 |
| **10ppb** |  | 100.00 |  | 100.00 |  | 100.00 |
| **0ppb** | **1** | 100.00 | **1** | 100.00 | **1** | 100.00 |
| **5 ppb** |  | 100.00 |  | 100.00 |  | 100.00 |
| **7 ppb** |  | 100.00 |  | 100.00 |  | 92.68 |
| **10ppb** |  | 100.00 |  | 100.00 |  | 100.00 |
| **0ppb** | **2** | 100.00 | **2** | 100.00 | **2** | 100.00 |
| **5 ppb** |  | 100.00 |  | 100.00 |  | 97.83 |
| **7 ppb** |  | 100.00 |  | 98.44 |  | 91.89 |
| **10ppb** |  | 97.53 |  | 100.00 |  | 100.00 |
| **0ppb** | **3** | 100.00 | **3** | 100.00 | **3** | 100.00 |
| **5 ppb** |  | 100.00 |  | 100.00 |  | 93.48 |
| **7 ppb** |  | 98.28 |  | 98.25 |  | 85.71 |
| **10ppb** |  | 93.51 |  | 98.53 |  | 96.77 |
| **0ppb** | **4** | 96.36 | **4** | 100.00 | **4** | 100 |
| **5 ppb** |  | 94.83 |  | 96.77 |  | 93.33 |
| **7 ppb** |  | 98.25 |  | 94.34 |  | 84.38 |
| **10ppb** |  | 91.43 |  | 96.67 |  | 96.00 |
| **0ppb** | **5** | 92.73 | **5** | 100.00 | **5** | 100.00 |
| **5 ppb** |  | 93.10 |  | 94.92 |  | 88.89 |
| **7 ppb** |  | 94.64 |  | 94.12 |  | 88.89 |
| **10ppb** |  | 92.42 |  | 97.67 |  | 91.67 |
| **0ppb** | **6** | 96.36 | **6** | 98.61 | **6** | 98.00 |
| **5 ppb** |  | 87.50 |  | 96.49 |  | 95.12 |
| **7 ppb** |  | 92.73 |  | 97.78 |  | 83.33 |
| **10ppb** |  | 85.25 |  | 96.88 |  | 85.71 |
| **0ppb** | **7** | 100 | **7** | 100.00 | **7** | 97.87 |
| **5 ppb** |  | 83.93 |  | 92.73 |  | 95.00 |
| **7 ppb** |  | 83.64 |  | 93.18 |  | 89.47 |
| **10ppb** |  | 83.64 |  | 92.31 |  | 78.95 |
