# Supplementary material for: One size does not fit all: Caste and sex differences in the response of bumblebees (Bombus impatiens) to chronic oral neonicotinoid exposure
Source: PLoS One. 2018 Oct 8;13(10):e0200041. doi: 10.1371/journal.pone.0200041 (PMC6175506; doi:10.1371/journal.pone.0200041)
Supplement: S3 Table — (DOCX) [file pone.0200041.s003.docx]

**S3 Table. List of clothianidin-induced non-detoxification genes with increased expression in worker (W) and male (M) *Bombus impatiens*.** Biological process known to be associated with each gene is also shown**.**

| **Gene ID** | **NCBI gene ID** | **P^a^** | **Fold change**  **W vs. M** | **Annotation** | **Biological process** |
| --- | --- | --- | --- | --- | --- |
| BIMP22468 | LOC100742232 | 0.01 | 3.17 | clavesin-2-like | lysosome organization in neuron |
| BIMP10861 | LOC100742053 | 0.02 | 3.08 | uncharacterized protein LOC100742053 |  |
| BIMP15244 | LOC100747227 | 0.02 | 2.18 | isopentenyl-diphosphate Delta-isomerase 1 | regulation of cholesterol biosynthetic processes |
| BIMP17595 | LOC100750162 | 0.02 | -2.59 | muscle-specific protein 20 | regulation of myoblast fusion |
| BIMP20364 | LOC100749090 | 0.02 | 2.87 | inhibin beta C chain | regulation of apoptosis/protein phosphorylation |
| BIMP16006 | LOC105681884 | 0.02 | 3.46 | uncharacterized LOC105681884 |  |
| BIMP17388 | LOC100740374 | 0.02 | -2.61 | anoctamin-4 isoform X2 | ion transmembrane transport |
| BIMP12048 | N/A | 0.03 | 1.99 | N/A |  |
| BIMP15690 | LOC100744117 | 0.03 | -2.68 | alpha-tocopherol transfer protein-like | response to toxic substance; lipid metabolic processes |
| BIMP16360 | LOC100748462 | 0.03 | 1.97 | RCC1 domain-containing protein 1 isoform X2 | cell cycle; transcription |
| BIMP19125 | LOC100740071 | 0.03 | -1.98 | lutropin-choriogonadotropic hormone receptor | reproduction |
| BIMP20180 | N/A | 0.03 | 2.43 | N/A |  |
| BIMP25024 | LOC100747881 | 0.03 | 1.91 | uncharacterized protein LOC100747881 |  |
| BIMP10379 | LOC100743319 | 0.03 | 2.42 | GTP-binding protein Di-Ras2 | signal transduction |
| BIMP25183 | LOC100741159 | 0.03 | 2.15 | exosome complex component RRP46 | immune defense |
| BIMP22395 | LOC100748872 | 0.04 | 2.01 | rho GTPase-activating protein 100F isoform X1 | signal transduction |
| BIMP23646 | LOC105680532 | 0.04 | 2.90 | uncharacterized LOC105680532 |  |
| BIMP10330 | LOC100742952 | 0.05 | -2.17 | protein vestigial isoform X2 | cell cycle;transcription |
| BIMP19854 | LOC100743072 | 0.05 | 1.66 | translocon-associated protein subunit gamma | SRP-dependent cotranslational protein targeting to membrane |
| BIMP17461 | LOC100747957 | 0.05 | 2.87 | metabotropic glutamate receptor 2 isoform X2 | synaptic transmission |
| BIMP15236 | LOC100740112 | 0.05 | 1.94 | reticulocyte-binding protein 2 homolog a-like isoform X1 | cell-cell adhesion |
| BIMP23054 | LOC100744620 | 0.05 | 1.96 | protein prickle-like | regulation of protein ubiquination |
| BIMP13268 | LOC100740371 | 0.06 | -1.60 | C-Maf-inducing protein-like | immune defense |
| BIMP15736 | LOC105680659 | 0.07 | -1.88 | muscle-specific protein 20-like | regulation of myoblast fusion |
| BIMP20006 | LOC100746509 | 0.07 | 1.71 | WD repeat-containing protein on Y chromosome-like | reproduction |
| BIMP24200 | LOC100747913 | 0.07 | -2.04 | uncharacterized protein LOC100747913 |  |
| BIMP16349 | LOC100746345 | 0.07 | 2.23 | protein Skeletor, isoforms B/C | cell division; meiosis |
| BIMP24694 | LOC100743725 | 0.07 | -1.64 | ankyrin repeat and BTB/POZ domain-containing protein BTBD11 isoform X3 | SMAD protein signal transduction |
| BIMP14873 | LOC100740376 | 0.07 | -1.56 | tubulin alpha-1 chain-like | cell division/cell cycle |
| BIMP18015 | N/A | 0.08 | -2.97 | N/A |  |
| BIMP18549 | LOC100745223 | 0.08 | 1.65 | uncharacterized protein LOC100745223 |  |
| BIMP10485 | LOC100741801 | 0.08 | -1.77 | uncharacterized protein LOC100741801 |  |
| BIMP22084 | LOC100743641 | 0.08 | -1.89 | titin isoform X3 | locomotion |
| BIMP25276 | LOC100747793 | 0.08 | -1.59 | serine/arginine repetitive matrix protein 2 isoform X2 | mRNA slicing |
| BIMP11842 | LOC100750030 | 0.08 | -1.57 | dnaJ homolog subfamily C member 8 | mRNA slicing in nervous system |
| BIMP22938 | LOC100740026 | 0.08 | 1.72 | histone H2A-like | chromatin silencing |
| BIMP14909 | LOC105680332 | 0.09 | 1.87 | uncharacterized protein C15orf61 homolog |  |
| BIMP21819 | LOC100749144 | 0.09 | 1.68 | 28S ribosomal protein S7 mitochondrial | translation |
| BIMP11483 | LOC100740114 | 0.09 | -2.39 | outer dense fiber protein 3-like | reproduction (spermatogenesis) |
| BIMP14510 | LOC100743860 | 0.09 | -1.80 | uncharacterized protein LOC100743860 |  |

^a^FDR adjusted p-value
