# Supplementary material for: One size does not fit all: Caste and sex differences in the response of bumblebees (Bombus impatiens) to chronic oral neonicotinoid exposure
Source: PLoS One. 2018 Oct 8;13(10):e0200041. doi: 10.1371/journal.pone.0200041 (PMC6175506; doi:10.1371/journal.pone.0200041)
Supplement: S4 Table — (DOCX) [file pone.0200041.s004.docx]

**S4 Table. List of clothianidin-induced genes showing a similar expression level in worker (W) and male (M) *Bombus impatiens*.**

| **Gene ID** | **NCBI gene ID** | **P^a^** | **Fold change (W vs. M)** | **Annotation** |
| --- | --- | --- | --- | --- |
| BIMP22020 | LOC100741771 | 0.10 | -1.56 | protein archease-like |
| BIMP11956 | LOC100749289 | 0.11 | -1.82 | kynurenine/alpha-aminoadipate aminotransferase mitochondrial-like |
| BIMP20886 | LOC100741841 | 0.12 | -1.45 | COP9 signalosome complex subunit 8 |
| BIMP20554 | LOC100742556 | 0.13 | -1.73 | glucose dehydrogenase [FAD, quinone]-like |
| BIMP14887 | LOC100745772 | 0.13 | -2.39 | glutathione S-transferase 1-1 |
| BIMP10283 | LOC100740351 | 0.13 | 1.47 | broad-complex core protein isoforms 1/2/3/4/5 |
| BIMP17539 | LOC100740253 | 0.14 | 1.91 | neuroendocrine convertase 1-like |
| BIMP11923 | LOC100748603 | 0.14 | 1.73 | 7-methylguanosine phosphate-specific 5'-nucleotidase |
| BIMP21692 | N/A | 0.14 | 1.77 | N/A |
| BIMP24100 | LOC100748348 | 0.14 | -2.14 | sestrin-1 |
| BIMP17695 | LOC100745261 | 0.14 | 2.13 | neuropeptide Y receptor type 2 |
| BIMP12343 | LOC100749760 | 0.16 | -1.54 | sodium channel protein para isoform X9 |
| BIMP23704 | LOC100743013 | 0.16 | 2.01 | sporulation-specific protein 15 isoform X2 |
| BIMP21529 | LOC100743536 | 0.16 | 1.48 | death-associated protein kinase 1-like isoform X1 |
| BIMP20448 | LOC105680950 | 0.16 | 1.46 | protein DGCR14 homolog |
| BIMP20363 | LOC100748409 | 0.17 | 1.98 | cathepsin L1-like |
| BIMP20463 | LOC100741979 | 0.17 | -1.78 | POC1 centriolar protein homolog A |
| BIMP14382 | LOC100747447 | 0.17 | 1.47 | uncharacterized protein LOC100747447 |
| BIMP16701 | N/A | 0.17 | -2.34 | N/A |
| BIMP18346 | LOC100745695 | 0.17 | 1.45 | cholinesterase transcript variant X3 |
| BIMP13907 | LOC100740050 | 0.17 | 1.46 | ribonuclease P protein subunit p25-like protein |
| BIMP20393 | LOC100745502 | 0.17 | 1.85 | dipeptidase 1 |
| BIMP25804 | LOC100750199 | 0.18 | 2.22 | uncharacterized protein LOC100750199 |
| BIMP11132 | LOC100746168 | 0.18 | 1.42 | sodium-dependent nutrient amino acid transporter 1-like |
| BIMP22299 | LOC100743635 | 0.18 | -1.83 | inosine-5'-monophosphate dehydrogenase 1b-like isoform X1 |
| BIMP15893 | LOC100739998 | 0.18 | -1.41 | AP-1 complex subunit mu-1-like |
| BIMP14951 | LOC100748598 | 0.19 | -1.73 | suppressor of lurcher protein 1 isoform X1 |
| BIMP23651 | LOC100741338 | 0.19 | 1.61 | 4-nitrophenylphosphatase |
| BIMP22419 | N/A | 0.19 | -1.58 | N/A |
| BIMP14942 | LOC100748477 | 0.19 | -2.49 | uncharacterized protein LOC100748477 |
| BIMP15490 | LOC100740830 | 0.20 | 1.38 | sorting nexin-27 isoform X1 |
| BIMP20816 | LOC105682087 | 0.20 | 2.00 | uncharacterized protein LOC105682087 |
| BIMP24815 | LOC100743804 | 0.20 | 1.62 | stress response protein NST1-like |
| BIMP17254 | LOC100749342 | 0.20 | 1.44 | glycerol kinase |
| BIMP24445 | LOC100741808 | 0.22 | 1.53 | graves disease carrier protein homolog isoform X1 |
| BIMP18541 | LOC100749258 | 0.22 | 1.40 | short-chain dehydrogenase/reductase family 16C member 6-like isoform X1 |
| BIMP10486 | LOC100741995 | 0.22 | -1.44 | succinate dehydrogenase [ubiquinone] flavoprotein subunit mitochondrial-like |
| BIMP20984 | LOC100748644 | 0.22 | 1.50 | protoporphyrinogen oxidase |
| BIMP15443 | LOC100746577 | 0.22 | 1.39 | uncharacterized protein C9orf117 homolog |
| BIMP13921 | LOC100746818 | 0.22 | 1.82 | lymphokine-activated killer T-cell-originated protein kinase |
| BIMP15412 | LOC100747603 | 0.22 | 1.49 | WD repeat-containing protein 35 isoform X2 |
| BIMP22872 | LOC100748967 | 0.24 | 1.61 | alanine--glyoxylate aminotransferase 2 mitochondrial isoform X1 |
| BIMP10656 | LOC100740384 | 0.24 | 1.36 | ATP synthase subunit s mitochondrial |
| BIMP14979 | LOC100742974 | 0.24 | 1.44 | cell division cycle-associated protein 7-like |
| BIMP25536 | LOC100746803 | 0.24 | 1.36 | laminin subunit beta-1 isoform X1 |
| BIMP11225 | LOC100745242 | 0.26 | 1.71 | probable G-protein coupled receptor Mth-like 1 |
| BIMP23060 | LOC100745908 | 0.26 | 1.49 | arf-GAP with dual PH domain-containing protein 1-like |
| BIMP16216 | LOC105680353 | 0.27 | 1.70 | solute carrier family 2 facilitated glucose transporter member 2-like |
| BIMP17915 | LOC100746827 | 0.27 | 1.42 | kelch-like protein 5 transcript variant X2 |
| BIMP21520 | LOC100744677 | 0.27 | 1.39 | uncharacterized protein LOC100744677 |
| BIMP21514 | LOC100744920 | 0.27 | 1.45 | Down syndrome cell adhesion molecule-like protein Dscam2 |
| BIMP19797 | LOC100748083 | 0.27 | 1.38 | bifunctional coenzyme A synthase |
| BIMP17838 | LOC100749499 | 0.27 | 1.39 | flavin-containing monooxygenase FMO GS-OX4-like isoform X1 |
| BIMP23719 | LOC100743702 | 0.30 | 1.30 | ATP-dependent RNA helicase DDX24 transcript variant X2 |
| BIMP11975 | LOC100750104 | 0.30 | -1.30 | nuclear migration protein nudC |
| BIMP20060 | LOC100747814 | 0.30 | 1.38 | AMME syndrome candidate gene 1 protein-like |
| BIMP24910 | LOC100750157 | 0.30 | 1.30 | high-affinity choline transporter 1-like isoform X1 |
| BIMP20073 | LOC100746894 | 0.30 | -1.47 | uncharacterized protein LOC100746894 |
| BIMP18767 | LOC100747302 | 0.31 | 1.75 | farnesol dehydrogenase-like |
| BIMP14257 | N/A | 0.32 | -1.46 | N/A |
| BIMP13423 | LOC100743706 | 0.32 | -1.30 | U4/U6 small nuclear ribonucleoprotein Prp4 |
| BIMP24845 | LOC100741639 | 0.33 | 1.34 | PI-PLC X domain-containing protein 3 isoform X1 |
| BIMP13337 | LOC100740523 | 0.33 | 1.39 | neurotrimin isoform X1 |
| BIMP11943 | LOC100746774 | 0.33 | 1.29 | F-actin-capping protein subunit beta isoform X2 |
| BIMP23821 | LOC100742661 | 0.33 | 1.31 | citron Rho-interacting kinase-like isoform X2 |
| BIMP23397 | LOC100740048 | 0.33 | 1.36 | uncharacterized protein LOC100740048 |
| BIMP15116 | LOC100741182 | 0.33 | 1.32 | cytochrome b5 isoform X4 |
| BIMP24697 | LOC100742966 | 0.33 | 1.59 | elongation of very long chain fatty acids protein 4-like |
| BIMP17382 | LOC100742293 | 0.33 | -1.45 | glyoxylate reductase/hydroxypyruvate reductase-like |
| BIMP13277 | LOC100748871 | 0.34 | 1.36 | innexin inx1 |
| BIMP13533 | LOC100742427 | 0.34 | -1.50 | protein Skeletor isoforms B/C isoform X1 |
| BIMP19485 | LOC100744124 | 0.34 | 1.29 | suppressor of fused homolog |
| BIMP17949 | LOC100745712 | 0.36 | 1.28 | zinc finger protein GLI4-like |
| BIMP14615 | LOC100740276 | 0.36 | 1.52 | protein claret segregational |
| BIMP22074 | LOC100744587 | 0.36 | 1.30 | acetyl-coenzyme A synthetase |
| BIMP25523 | LOC100744836 | 0.36 | -1.32 | DNA-binding protein RFXANK |
| BIMP23268 | LOC100745353 | 0.36 | -1.32 | FMRFamide receptor-like |
| BIMP20637 | LOC100743367 | 0.37 | 1.25 | alpha-tocopherol transfer protein isoform X1 |
| BIMP19490 | LOC100745369 | 0.38 | 1.37 | sperm-associated antigen 1 |
| BIMP17589 | N/A | 0.38 | 1.72 | N/A |
| BIMP13338 | LOC100740523 | 0.38 | 1.40 | neurotrimin transcript variant X1 |
| BIMP12022 | LOC100744356 | 0.38 | -1.27 | REST corepressor 3 isoform X1 |
| BIMP12510 | N/A | 0.38 | 1.40 | N/A |
| BIMP17380 | LOC100742775 | 0.38 | 1.24 | uncharacterized protein LOC100742775 isoform X1 |
| BIMP18939 | LOC100744454 | 0.38 | -1.39 | NEDD8 |
| BIMP20321 | LOC100748698 | 0.38 | -1.34 | speckle targeted PIP5K1A-regulated poly(A) polymerase-like |
| BIMP18867 | LOC100750203 | 0.38 | 1.34 | uncharacterized protein C14orf119 |
| BIMP13669 | LOC100743545 | 0.38 | -1.25 | WD repeat-containing protein 47 isoform X3 |
| BIMP15710 | LOC100745684 | 0.39 | 1.31 | rhomboid-related protein 4-like |
| BIMP25518 | LOC100744161 | 0.39 | 1.22 | ephrin type-A receptor 3-like |
| BIMP13582 | LOC100742282 | 0.39 | -1.30 | sodium/calcium exchanger 3 |
| BIMP18086 | LOC100744460 | 0.39 | -1.51 | venom protease |
| BIMP19979 | LOC100746985 | 0.39 | -1.44 | uncharacterized protein LOC100746985 |
| BIMP20474 | LOC100750156 | 0.39 | 1.27 | SH2B adapter protein 1 isoform X1 |
| BIMP13802 | LOC105681035 | 0.39 | -1.48 | uncharacterized protein LOC105681035 |
| BIMP22318 | LOC100747340 | 0.39 | -1.31 | alpha-endosulfine |
| BIMP25206 | LOC100749141 | 0.39 | 1.27 | tRNA-specific adenosine deaminase 2-like, transcript variant X1, mRNA |
| BIMP10857 | LOC100742823 | 0.39 | 1.30 | uncharacterized protein LOC100742823 isoform X2 |
| BIMP12288 | LOC100741063 | 0.39 | 1.51 | uncharacterized LOC100741063 |
| BIMP16419 | LOC100745964 | 0.39 | 1.25 | rRNA 2'-O-methyltransferase fibrillarin |
| BIMP16180 | LOC100742378 | 0.39 | -1.56 | SET and MYND domain-containing protein 4-like isoform X2 |
| BIMP23515 | LOC100740075 | 0.39 | 1.79 | uncharacterized LOC100740075 |
| BIMP25455 | LOC100740930 | 0.40 | -1.30 | uncharacterized LOC100740930 |
| BIMP18521 | LOC100743234 | 0.40 | 1.32 | nicotinamide riboside kinase 1 |
| BIMP13512 | LOC100749171 | 0.40 | -1.27 | 46 kDa FK506-binding nuclear protein |
| BIMP11241 | LOC105681917 | 0.40 | 1.26 | protein tesmin/TSO1-like CXC 3 |
| BIMP24544 | LOC100744399 | 0.40 | 1.30 | uncharacterized protein F58A4.6 |
| BIMP20971 | LOC100749562 | 0.40 | -1.61 | feline leukemia virus subgroup C receptor-related protein 2-like |
| BIMP16878 | LOC100742767 | 0.40 | 2.31 | single-minded homolog 2 isoform X3 |
| BIMP23923 | LOC100747006 | 0.40 | -1.21 | LIM and senescent cell antigen-like-containing domain protein 1 isoform X1 |
| BIMP14401 | LOC100741102 | 0.40 | 1.35 | spindle and kinetochore-associated protein 1-like isoform X1 |
| BIMP24824 | LOC100745873 | 0.40 | 1.46 | uncharacterized protein LOC100745873 |
| BIMP24136 | LOC100742409 | 0.40 | -1.30 | zinc finger protein 271-like |
| BIMP23711 | LOC100746450 | 0.40 | -1.33 | S phase cyclin A-associated protein in the endoplasmic reticulum transcript variant X1 |
| BIMP25743 | LOC100746518 | 0.41 | -1.21 | sn1-specific diacylglycerol lipase alpha isoform X3 |
| BIMP21312 | LOC100749404 | 0.41 | 1.21 | POU domain class 2 transcription factor 1-like isoform X2 |
| BIMP13817 | LOC100745057 | 0.41 | 1.25 | eukaryotic translation initiation factor 4E type 2 isoform X3 |
| BIMP18858 | LOC100744455 | 0.41 | -1.22 | uncharacterized protein C1orf43 homolog |
| BIMP21359 | LOC100740214 | 0.41 | 1.22 | tubulin beta chain-like isoform X2 |
| BIMP20739 | LOC100745831 | 0.43 | -1.24 | synaptosomal-associated protein 25 isoform X2 |
| BIMP10577 | LOC100741178 | 0.44 | 1.28 | pancreatic triacylglycerol lipase-like |
| BIMP14412 | LOC100742864 | 0.44 | 1.22 | adenomatous polyposis coli protein-like isoform X1 |
| BIMP22297 | LOC100741390 | 0.44 | 1.27 | pyroglutamyl-peptidase 1 |
| BIMP20608 | LOC100743968 | 0.44 | -1.22 | prolactin regulatory element-binding protein |
| BIMP13787 | LOC100740639 | 0.44 | -1.20 | uncharacterized protein LOC100740639 isoform X1 |
| BIMP10524 | LOC100749158 | 0.46 | -1.32 | sister chromatid cohesion protein DCC1 |
| BIMP21194 | LOC105680565 | 0.46 | -1.22 | EF-hand domain-containing protein 1-like |
| BIMP25337 | LOC100743486 | 0.46 | 1.28 | glutathione S-transferase theta-1-like |
| BIMP13130 | LOC100749113 | 0.46 | 1.44 | COMM domain-containing protein 5-like |
| BIMP16240 | LOC100745367 | 0.47 | -1.03 | uncharacterized protein LOC100745367 |
| BIMP16316 | LOC100749297 | 0.47 | -1.35 | growth arrest-specific protein 8 |
| BIMP16600 | LOC100744069 | 0.48 | 1.28 | CUGBP Elav-like family member 2 |
| BIMP17292 | LOC100745766 | 0.48 | 1.28 | uncharacterized protein C45G9.7 |
| BIMP22575 | LOC100742606 | 0.48 | 1.19 | putative acyl-CoA-binding protein |
| BIMP25865 | LOC100749960 | 0.49 | -1.22 | autophagy protein 5 |
| BIMP11283 | N/A | 0.49 | -1.24 | N/A |
| BIMP23031 | LOC100740151 | 0.49 | -1.27 | histone H2B-like |
| BIMP15212 | LOC100745874 | 0.49 | 1.21 | heparin sulfate O-sulfotransferase isoform X3 |
| BIMP16880 | LOC100743576 | 0.50 | -1.23 | proto-oncogene tyrosine-protein kinase ROS |
| BIMP22042 | LOC100749662 | 0.50 | -1.35 | matrix metalloproteinase-24-like isoform X1 |
| BIMP12105 | N/A | 0.50 | -1.25 | N/A |
| BIMP13580 | LOC100742530 | 0.53 | 1.15 | leucine-rich repeat-containing protein 70 |
| BIMP22873 | LOC100743262 | 0.53 | 1.20 | VWFA and cache domain-containing protein 1 |
| BIMP22112 | LOC100746871 | 0.56 | -1.18 | E3 ubiquitin-protein ligase FANCL isoform X1 |
| BIMP22426 | LOC100744638 | 0.57 | 1.18 | U1 small nuclear ribonucleoprotein C |
| BIMP18145 | LOC100741717 | 0.58 | 1.14 | electron transfer flavoprotein subunit alpha mitochondrial |
| BIMP21272 | LOC100740582 | 0.58 | 1.16 | vacuolar protein sorting-associated protein 28 homolog |
| BIMP22976 | LOC105681117 | 0.59 | -1.18 | uncharacterized LOC105681117 |
| BIMP16605 | LOC100743353 | 0.59 | -1.15 | uncharacterized protein LOC100743353 |
| BIMP24438 | LOC100742616 | 0.59 | 1.16 | GTPase-activating Rap/Ran-GAP domain-like protein 3 isoform X3 |
| BIMP22728 | LOC100746979 | 0.59 | 1.19 | phospholipid-transporting ATPase |
| BIMP17512 | LOC100749555 | 0.60 | 1.14 | splicing factor 3B subunit 4 |
| BIMP18460 | LOC105680195 | 0.60 | 1.16 | nuclear pore complex protein Nup205 isoform X1 |
| BIMP15664 | LOC100742611 | 0.61 | -1.34 | uncharacterized LOC100742611 |
| BIMP13796 | LOC100742284 | 0.61 | 1.23 | Bombus impatiens S-adenosylmethionine decarboxylase proenzyme (LOC100742284), transcript variant X1, mRNA |
| BIMP25071 | LOC100746825 | 0.61 | 1.21 | carboxypeptidase N subunit 2-like |
| BIMP12271 | LOC100746970 | 0.61 | 1.17 | replication protein A 32 kDa subunit-like isoform X2 |
| BIMP16938 | LOC100742318 | 0.61 | 1.15 | band 4.1-like protein 4 isoform X2 |
| BIMP17392 | LOC100749349 | 0.61 | -1.14 | putative fatty acyl-CoA reductase CG5065 |
| BIMP25866 | LOC100746106 | 0.61 | 1.14 | nose resistant to fluoxetine protein 6 |
| BIMP12419 | LOC100747969 | 0.61 | -1.25 | doublesex- and mab-3-related transcription factor A2 isoform X1 |
| BIMP19170 | LOC100744983 | 0.61 | 1.21 | leucine-rich PPR motif-containing protein mitochondrial |
| BIMP18006 | LOC100747506 | 0.62 | -1.27 | probable G-protein coupled receptor No9 |
| BIMP17346 | N/A | 0.62 | 1.23 | N/A |
| BIMP24219 | LOC100743470 | 0.62 | 1.13 | uncharacterized protein LOC100743470 isoform X2 |
| BIMP21171 | LOC100745530 | 0.62 | 1.21 | protein Mpv17 |
| BIMP23438 | LOC100743046 | 0.62 | -1.21 | succinate dehydrogenase assembly factor 3 mitochondrial |
| BIMP15691 | LOC100742731 | 0.62 | 1.16 | rho-related BTB domain-containing protein 1 isoform X2 |
| BIMP25333 | LOC100742008 | 0.64 | 1.12 | sialin |
| BIMP24912 | LOC100749599 | 0.66 | -1.14 | uncharacterized protein LOC100749599 isoform X3 |
| BIMP13308 | LOC100741893 | 0.66 | 1.15 | intraflagellar transport protein 74 homolog isoform X1 |
| BIMP13903 | LOC105680747 | 0.66 | -1.13 | cGMP-dependent protein kinase 1 isoform X1 |
| BIMP18994 | N/A | 0.66 | 1.22 | N/A |
| BIMP24446 | LOC100741448 | 0.66 | 1.13 | TATA box-binding protein-like protein 1 |
| BIMP13738 | N/A | 0.66 | 1.30 | N/A |
| BIMP13804 | LOC100744191 | 0.66 | 1.13 | jouberin-like isoform X1 |
| BIMP15569 | LOC100741282 | 0.66 | -1.12 | sodium-dependent phosphate transport protein 1 chloroplastic-like isoform X1 |
| BIMP23565 | LOC100743404 | 0.67 | 1.26 | zinc finger protein 808 |
| BIMP10487 | LOC100742115 | 0.67 | -1.12 | succinate dehydrogenase [ubiquinone] flavoprotein subunit mitochondrial |
| BIMP10013 | LOC100741439 | 0.67 | 1.16 | vascular endothelial growth factor receptor 1 transcript variant X2 |
| BIMP22656 | LOC100745901 | 0.67 | 1.17 | uncharacterized protein LOC100745901 |
| BIMP13576 | LOC100742999 | 0.68 | 1.13 | pre-mRNA-splicing factor ISY1 homolog |
| BIMP16788 | LOC105680674 | 0.69 | 1.33 | uncharacterized protein LOC105680674 |
| BIMP21342 | LOC100749356 | 0.71 | 1.08 | serine/threonine-protein kinase Warts |
| BIMP15438 | LOC100744810 | 0.72 | -1.11 | D-3-phosphoglycerate dehydrogenase |
| BIMP14897 | LOC100744168 | 0.72 | 1.13 | cyclin-dependent kinase 2-like |
| BIMP14327 | LOC100748457 | 0.72 | -1.10 | anaphase-promoting complex subunit 10 |
| BIMP20072 | LOC100747015 | 0.72 | -1.37 | Bombus impatiens uncharacterized LOC100747015 |
| BIMP22944 | LOC100749450 | 0.72 | 1.12 | alpha/beta hydrolase domain-containing protein 13 |
| BIMP13330 | LOC100749869 | 0.73 | 1.14 | peripheral plasma membrane protein CASK transcript variant X1 |
| BIMP13303 | LOC100740161 | 0.74 | 1.11 | putative peptidyl-prolyl cis-trans isomerase dodo |
| BIMP13863 | LOC100744257 | 0.74 | -1.14 | cysteine-rich protein 1-like |
| BIMP20344 | LOC105680867 | 0.75 | -1.38 | aquaporin-11 isoform X1 |
| BIMP11574 | LOC100749886 | 0.75 | 1.10 | probable 39S ribosomal protein L23 mitochondrial |
| BIMP24722 | LOC100744395 | 0.75 | -1.08 | putative N-acetylglucosamine-6-phosphate deacetylase |
| BIMP23616 | LOC100742809 | 0.75 | -1.19 | L-threonine 3-dehydrogenase mitochondrial isoform X1 |
| BIMP15720 | LOC100747952 | 0.75 | -1.10 | zinc transporter 1 isoform X1 |
| BIMP10869 | LOC100742464 | 0.75 | 1.12 | uncharacterized protein LOC100742464 |
| BIMP25696 | LOC100743879 | 0.75 | 1.09 | phosphatidate cytidylyltransferase mitochondrial |
| BIMP16311 | LOC100749533 | 0.75 | 1.11 | PRADC1-like protein |
| BIMP24466 | LOC100747751 | 0.76 | -1.07 | N-acetylneuraminate lyase-like |
| BIMP16759 | LOC100749044 | 0.76 | -1.16 | LIM and SH3 domain protein Lasp isoform X2 |
| BIMP25504 | LOC100743209 | 0.76 | -1.09 | charged multivesicular body protein 6 |
| BIMP20567 | LOC100742326 | 0.77 | 1.09 | uncharacterized protein LOC100742326 |
| BIMP17258 | LOC100748558 | 0.77 | -1.08 | actin-binding protein IPP |
| BIMP10011 | LOC100741439 | 0.78 | 1.22 | vascular endothelial growth factor receptor 1 transcript variant X2;transcript |
| BIMP20312 | LOC100742752 | 0.79 | 1.08 | semaphorin-1A-like transcript variant X3 |
| BIMP22021 | LOC100741567 | 0.80 | -1.13 | transcription factor E2F2 isoform X1 |
| BIMP17024 | LOC100749337 | 0.80 | -1.09 | calmodulin |
| BIMP24125 | LOC100747309 | 0.80 | 1.11 | adenylate kinase isoenzyme 5 transcript variant X3 |
| BIMP13632 | LOC105680466 | 0.80 | 1.11 | SOSS complex subunit B homolog |
| BIMP17195 | LOC100742459 | 0.80 | 1.09 | glyoxylate reductase/hydroxypyruvate reductase |
| BIMP15188 | LOC105682047 | 0.80 | -1.13 | uncharacterized protein LOC105682047 |
| BIMP15122 | LOC100741780 | 0.81 | -1.07 | Krueppel-like factor 10 |
| BIMP14250 | LOC100746058 | 0.82 | 1.15 | facilitated trehalose transporter Tret1 |
| BIMP10030 | LOC100742600 | 0.82 | -1.06 | protein MAK16 homolog A |
| BIMP25581 | LOC100740396 | 0.82 | 1.11 | exonuclease 1 isoform X2 |
| BIMP21487 | LOC100741718 | 0.83 | -1.15 | DDB1- and CUL4-associated factor 7 |
| BIMP25108 | LOC100748899 | 0.83 | -1.12 | hexamerin-like |
| BIMP21426 | LOC100745771 | 0.83 | -1.07 | venom metalloproteinase 3 |
| BIMP10353 | LOC100749334 | 0.83 | -1.11 | uncharacterized protein LOC100749334 |
| BIMP10533 | LOC100742743 | 0.84 | 1.09 | uncharacterized protein LOC100742743 |
| BIMP10254 | LOC100745465 | 0.86 | 1.15 | scavenger receptor class B member 1-like |
| BIMP11339 | LOC100747231 | 0.86 | 1.07 | ras GTPase-activating protein-binding protein 2 |
| BIMP24217 | LOC100741910 | 0.86 | 1.08 | mpv17-like protein isoform X2 |
| BIMP10786 | LOC100740140 | 0.88 | 1.04 | lysine-specific histone demethylase 1A |
| BIMP11139 | LOC100745446 | 0.88 | 1.04 | eukaryotic translation initiation factor 3 subunit A-like (LOC100745446), |
| BIMP18816 | LOC100746269 | 0.88 | 1.04 | putative uncharacterized protein DDB_G0282133 |
| BIMP20658 | LOC100746035 | 0.88 | 1.12 | relaxin receptor 1 |
| BIMP19546 | LOC100749241 | 0.88 | -1.07 | uncharacterized protein LOC100749241 |
| BIMP22087 | LOC100743762 | 0.88 | -1.05 | zinc carboxypeptidase-like |
| BIMP23034 | LOC100748016 | 0.88 | 1.04 | ATP-binding cassette sub-family G member 1 |
| BIMP21017 | LOC100743794 | 0.89 | -1.06 | zinc transporter ZIP3 |
| BIMP23019 | LOC100743355 | 0.90 | 1.05 | cationic amino acid transporter 3 isoform X1 |
| BIMP15970 | LOC100749966 | 0.90 | -1.11 | gustatory receptor for sugar taste 64f-like |
| BIMP22551 | LOC100749382 | 0.91 | -1.03 | DNA-binding protein RFX2 isoform X2 |
| BIMP20104 | LOC100742670 | 0.91 | 1.04 | centromere-associated protein E-like |
| BIMP22892 | LOC105680800 | 0.92 | 1.05 | dynactin subunit 5 |
| BIMP24034 | LOC100750004 | 0.92 | -1.02 | bifunctional ATP-dependent dihydroxyacetone kinase/FAD-AMP lyase (cyclizing)-like |
| BIMP10291 | N/A | 0.92 | 1.08 | N/A |
| BIMP16594 | LOC100749331 | 0.92 | -1.05 | glycine dehydrogenase (decarboxylating) mitochondrial |
| BIMP24099 | LOC100748469 | 0.92 | -1.08 | hexokinase type 2-like |
| BIMP11032 | LOC100748895 | 0.92 | 1.01 | dedicator of cytokinesis protein 7 |
| BIMP22700 | LOC100740625 | 0.92 | -1.03 | sushi von Willebrand factor type A, EGF and pentraxin domain-containing protein 1-like |
| BIMP13240 | LOC100746841 | 0.92 | -1.17 | lipid storage droplets surface-binding protein 1 isoform X1 |
| BIMP20479 | LOC100741192 | 0.92 | 1.03 | ran-specific GTPase-activating protein-like |
| BIMP18364 | LOC100742779 | 0.92 | 1.02 | potassium voltage-gated channel protein eag transcript variant X7 |
| BIMP12097 | LOC100744989 | 0.93 | -1.06 | protein APCDD1-like |
| BIMP22921 | LOC100743230 | 0.93 | -1.05 | lachesin-like isoform X2 |
| BIMP19690 | LOC100745959 | 0.93 | -1.04 | ubiquitin carboxyl-terminal hydrolase 48-like isoform X1 |
| BIMP11227 | LOC100745484 | 0.93 | 1.03 | protein-tyrosine sulfotransferase |
| BIMP16924 | N/A | 0.93 | -1.09 | N/A |
| BIMP14324 | LOC100740263 | 0.93 | -1.05 | NADH dehydrogenase [ubiquinone] 1 alpha subcomplex subunit 9 mitochondrial |
| BIMP20601 | LOC105680205 | 0.94 | -1.01 | nuclear pore complex protein Nup93-like |
| BIMP23822 | LOC100742781 | 0.95 | 1.02 | fatty acid synthase |

^a^FDR adjusted p-value
